# Anti-icing performance on aluminum surfaces and proposed model for freezing time calculation

**DOI:** 10.1038/s41598-020-80886-x

**Published:** 2021-02-11

**Authors:** Van-Huy Nguyen, Ba Duc Nguyen, Hien Thu Pham, Su Shiung Lam, Dai-Viet N. Vo, Mohammadreza Shokouhimehr, Thi Hong Hanh Vu, Thanh-Binh Nguyen, Soo Young Kim, Quyet Van Le

**Affiliations:** 1 Department for Management of Science and Technology Development, Ton Duc Thang University, Ho Chi Minh City, Vietnam; 2 Faculty of Applied Sciences, Ton Duc Thang University, Ho Chi Minh City, Vietnam; 3 Basic Science Department, Tan Trao University, Tuyen Quang, Vietnam; 4 Surface Analysis Department, Samsung Display Vietnam, Bac Ninh, Vietnam; 5 Pyrolysis Technology Research Group, Higher Institution Centre of Excellence (HICoE), Institute of Tropical Aquaculture and Fisheries (AKUATROP), Universiti Malaysia Terengganu, 21030 Kuala Terengganu, Terengganu Malaysia; 6 Center of Excellence for Green Energy and Environmental Nanomaterials (CE@GrEEN), Nguyen Tat Thanh University, 300A Nguyen Tat Thanh, District 4, Ho Chi Minh City, 755414 Vietnam; 7 Department of Materials Science and Engineering, Research Institute of Advanced Materials, Seoul National University, Seoul, 08826 Republic of Korea; 8 Physics Faculty, Thai Nguyen University of Education, Thai Nguyen, Vietnam; 9 Department of Materials Science and Engineering, Institute of Green Manufacturing Technology, Korea University, 145 Anam-ro, Seongbuk-gu, Seoul, 02841 Republic of Korea; 10 Institute of Research and Development, Duy Tan University, Da Nang, 550000 Vietnam

**Keywords:** Engineering, Materials science

## Abstract

In this work, we proposed a facile approach to fabricate a superhydrophobic surface for anti-icing performance in terms of adhesive strength and freezing time. A hierarchical structure was generated on as-received Al plates using a wet etching method and followed with a low energy chemical compound coating. Surfaces after treatment exhibited the great water repellent properties with a high contact angle and extremely low sliding angle. An anti-icing investigation was carried out by using a custom-built apparatus and demonstrated the expected low adhesion and freezing time for icephobic applications. In addition, we proposed a model for calculating the freezing time. The experimented results were compared with theoretical calculation and demonstrated the good agreement, illustrating the importance of theoretical contribution in design icephobic surfaces. Therefore, this study provides a guideline for the understanding of icing phenomena and designing of icephobic surfaces.

## Introduction

Icing problems present many challenges as the diversity of ice formation. In natural environments, ice accumulation can be found on a wide range of temperatures and humidity owing to the different scenarios, including freezing rain, snow, and frost formation. Specifically, ice accretion on the wings of aircraft by freezing rain or fog icing may cause a sudden loss of control owing to the weight overloading and lack of lifting force. Moreover, ice bulks form on the fuselage may be ingested into the engines causing a partial or total loss of thrust ^1,2^. Furthermore, ice accretion on power transmission systems, vehicles, or offshore platforms might lead to massive damage and potentially endangering people ^3–5^. Generally, anti-icing strategies might be separated into active and passive methods. The current active strategies for combatting icing problems primarily involve the heating systems, chemical deicing fluids, and mechanical removal ^6–11^. On the opposite side, it would be advantageous if surfaces can passively hinder the ice formation and ensure the ease removal process without any external energy ^12–19^. These processes are more efficient, environmentally favorable compared to industrial active methods and can be achieved using the physicochemical process based on texturing structure incorporates with a low-surface-energy compound.

Superhydrophobic surfaces, which inspired by the Lotus leaf concept, are believed as a promising strategy for anti-icing materials owing to their water repellent ^20–23^. Many types of research have reported the efficient passive anti-icing methods using a superhydrophobic phenomenon for reducing adhesion force ^24–32^ or delaying freezing time ^33–35^. In this study, we critically examined the facile strategy for attaining icephobicity on the superhydrophobic surface and compared it with a wide range of wettability to point out the effect of surface energy in anti-icing performance. The micro-nano hierarchical structure was generated on as-received Al plate through wet etching and followed by a low surface energy material coating to enable a perfect water repellent surface and nucleation inhibitor as well. Experimental results were compared with ongoing researches to figure out the contribution of surface wettability on anti-icing performance. Also, we proposed a calculation model to determine the freezing time. Freezing time experiments carried out on all samples were compared with theoretical calculation and revealed a good agreement, demonstrating the appropriate model for designing icephobic surfaces. The originality of this work is the experimental demonstration of the anti-icing performance on the superhydrophobic surface and proposing comprehensive insight into icing phenomena for icephobic applications.

## Results and discussion

The ability to imitate the lotus leaf micro-nano structure enables the manufacturing of a superhydrophobic surface with extraordinary water repellence. The superhydrophobic concept, therefore, has been widely developed for icephobic applications owing to its beneficial properties including drag reduction and self-cleaning ability. However, recent studies on textured superhydrophobic surfaces have revealed that the performance largely limited by environmental constraints while the system cannot prevent the ice nucleation or obstruct the frost accumulation on surface textures. Besides, the voids between surface features might serve as vulnerabilities under extreme humidity conditions, results in the interlocking effect and eventually comparable adhesion strength on superhydrophobic and superhydrophilic substrates.

**Figure 1:**
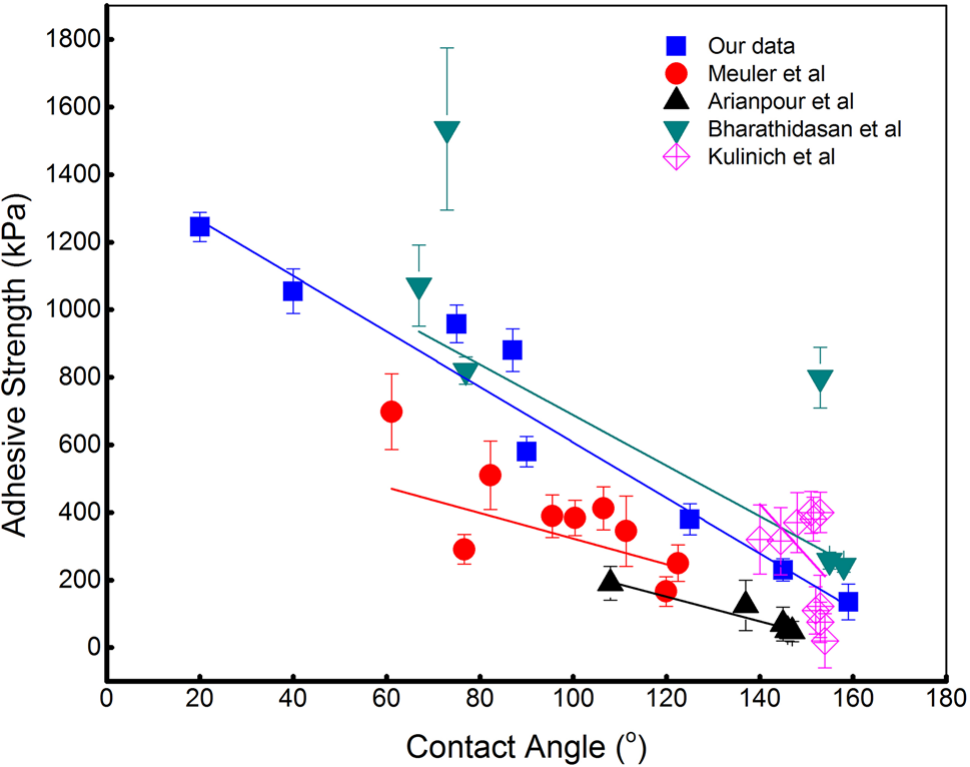
The original version of Figure 8.

To basically investigate the contribution of wettability in anti-icing performance, a wide range of contact angles was examined against the corresponded adhesive strength. Figure 1 disclosures the linear relation between surface wettability and adhesive strength. Our data were also compared with some ongoing research with the same interest and indicated the relatively same tendency as the higher contact angle sample exhibited the significant low adhesion compared to higher surface energy samples. The lowest value belongs to the superhydrophobic sample with about 135 kPa, which is % lower than 145° sample and about 9 times lower when compared with the superhydrophilic surface. The reason can be attributed to the contact area between ice and surface as the adhesion strength is attributed to the electrostatic force between molecules at the interface. Hence, the lower the contact area we can achieve, the lower the adhesion we can have. The measured adhesive strength gradually increases as the decrease of water-surface apparent contact angle.

**Figure 2:**
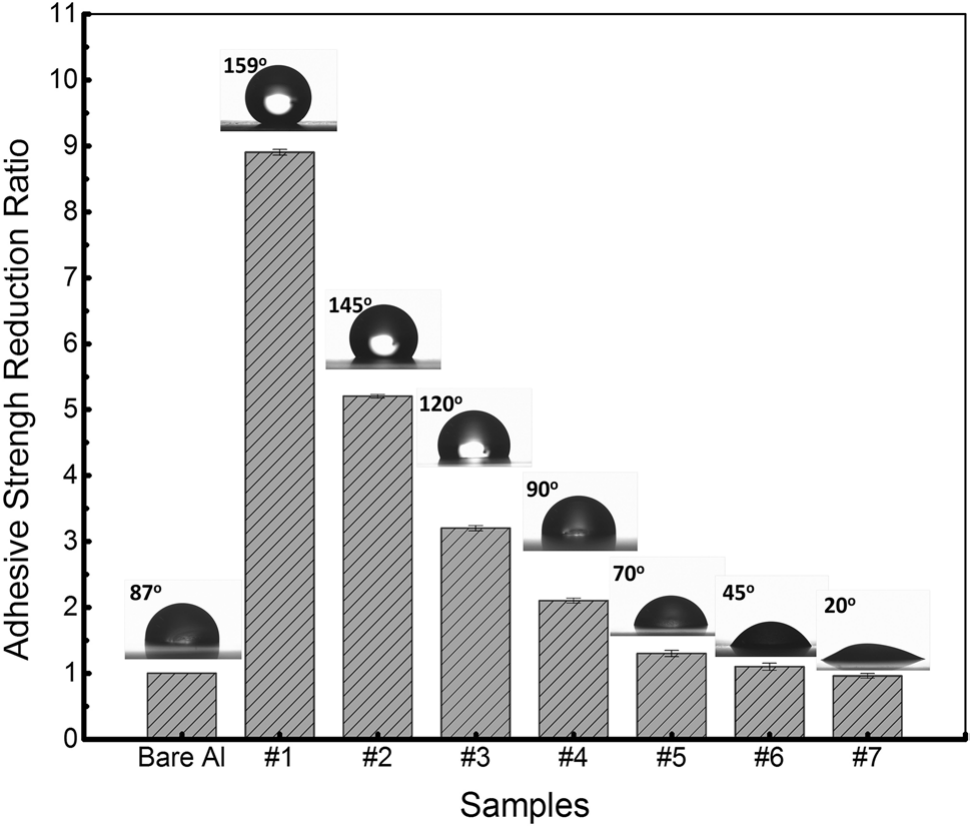
The reduction ratio of adhesion strength to as-received Al.

The advantages of the textured structure also can be emphasized through the reduction ratio (Fig. 2). The measurement on treated samples was compared to the one on as-received Al plate and essentially demonstrates the necessity of surface functionalization process for passive anti-icing applications. Almost textured samples propose a high reduction ratio even maintain a high contact area than the reference sample. The higher the contact angle we can yield, the higher the reduction ratio we can achieve. The higher surface energy i.e. lower contact angle corresponds to the higher affinity for water, finally results in the spreading form of a water droplet on the surface instead of forming a like-spherical droplet. This formation maintains in the whole freezing process so it eventually leads to higher ice-surface contact area. These results once again reinforced the importance of contact area in optimizing anti-icing effectiveness for icephobic applications. It should be noted here that the contact area parameter only viewed as an index factor when we consider surfaces with the same coating material.

**Figure 3:**
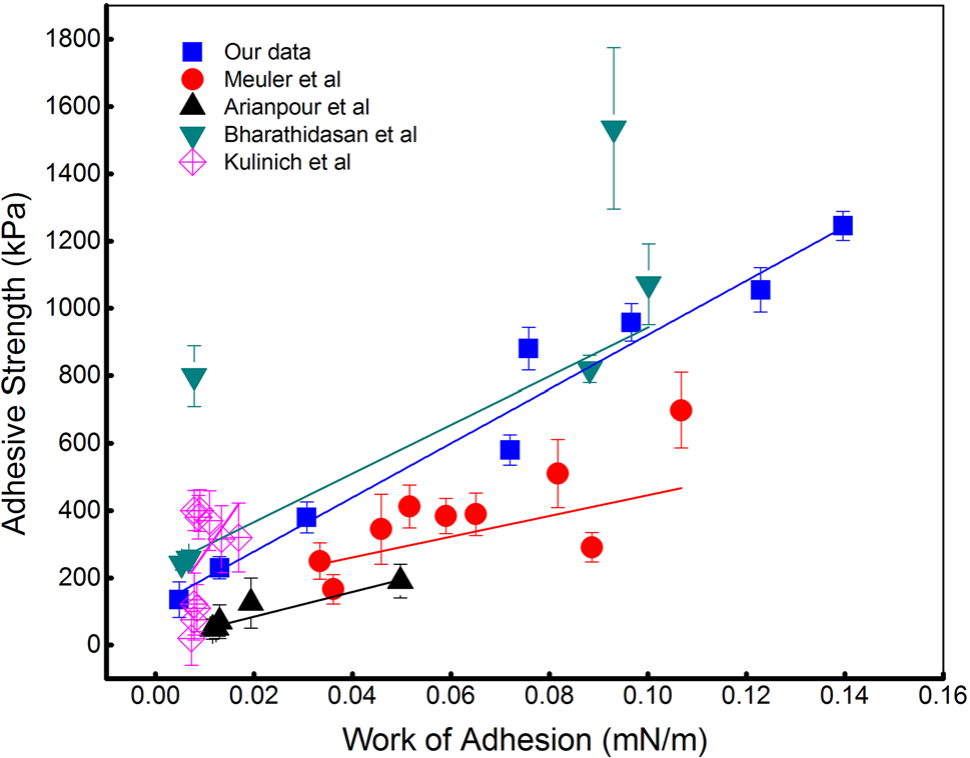
The work of adhesion in correlation with adhesive strength.

Ice often accumulates on a surface when water comes in contact with the surface that is at temperatures below the freezing point. This process consumes the energy, hence it is worth considering the work of the adhesion parameter, which refers to the work that must be done to separate two adjacent phases. Figure 3 shows the distribution of adhesive strength against the work of adhesion calculated from our data and relevant research. The tendency also demonstrates the linear correlation between two investigated terms and once essentially proves the guiding role of the work of adhesion parameters in anti-icing applications. It should be noted here that the discussed results were collected from different experiments working on disparate types of materials including polymer, aluminum, copper but surprisingly illustrated the same tendency.

**Figure 4:**
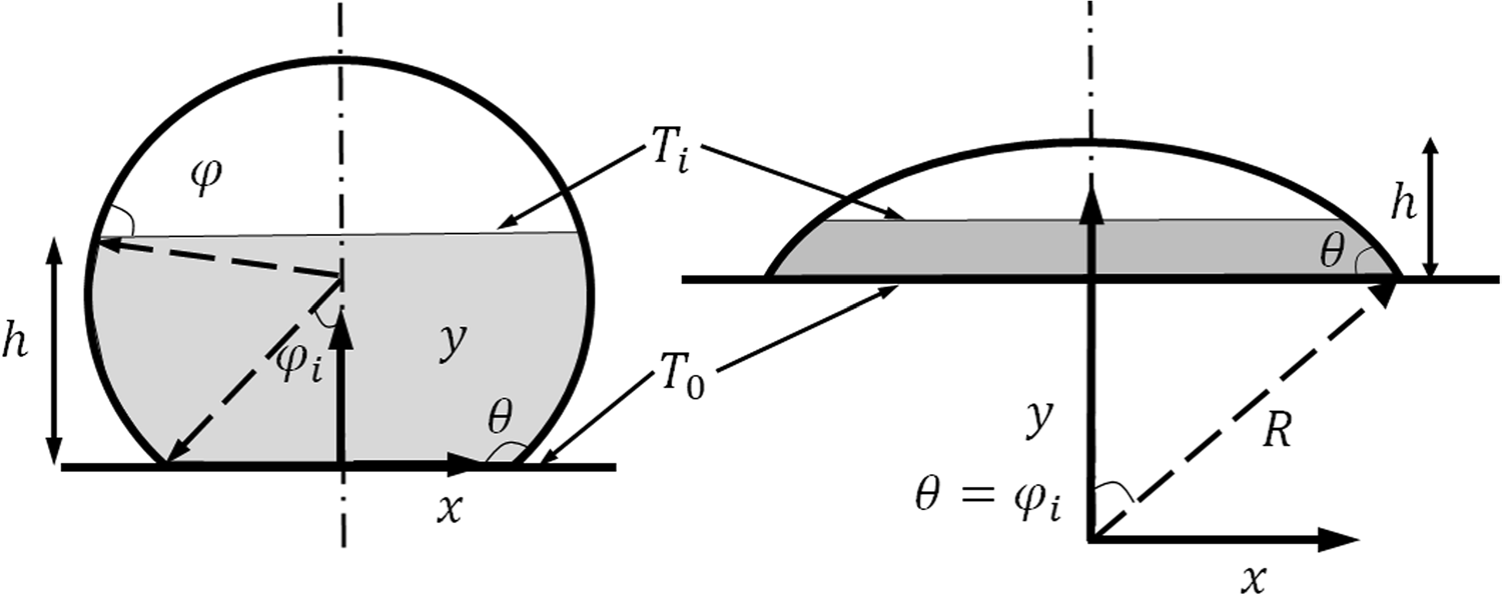
The theoretical model of a water droplet with a contact angle higher than 90° (left), and lower than 90° (right).

It is well known that the freezing process occurs by heat transfer from water volume to the cold substrate. However, to our best knowledge, there was no theoretical model to specifically predict the freezing time for an anti-icing experiment. In this work, we proposed a heat transfer model that aimed to investigate the whole freezing process and determine the freezing time. For ease understanding, we assume that water droplet forming on a sub-temperature surface in like-spherical shape in Fig. 4. We first begin by considering freezing a drop on a flat surface.

When the drop is placed on a flat and sub-cooled surface as shown in Fig. 4, the water drop starts to freeze from the contact region and the ice-water interface propagates until the water above it changes to ice. Here, the heat transfer rate *q* from the interface to the flat surface is

1 $$q = \mathop \int \limits_{0}^{h} k\Delta T\frac{A}{dh} = k\Delta T\left( {\mathop \int \limits_{0}^{h} \frac{1}{A}{\text{d}}y} \right)^{ - 1}$$

where $\Delta T = T_{i} - T_{s}$, *k* is the conductive coefficient of ice, and *A* is the interface area at the *dt* examined moment. They can be defined as

$$A = \pi r^{2} = \pi R^{2} \sin^{2} \varphi ;\quad dy = R\sin \varphi d\varphi ;\quad R = \frac{d}{\sin \theta }$$

Because of the heat transfer from the air to water during freezing is so small due to low heat transfer coefficient of air, heat transfer rate *q* will induce the phase change of the water above the interface to solid-phase:

2 $$q = \rho AL\frac{{{\text{d}}h}}{{{\text{d}}t}}$$

With $dh = \tan \varphi dr$, $\rho$ is the density of ice, *L* is the coefficient of latent heat of fusion, and *h* is the position of the water–ice interface from the substrate base.

Combining Eq. (1) and (2) leads to the freezing time of a drop on a surface:

3 $$dt = \frac{\rho AL}{{k\Delta T}}\mathop \int \limits_{0}^{h} \frac{1}{A}dydh$$

Then we have

4 $$t\frac{k\Delta T}{{\rho Ld}} = \mathop \int \limits_{\pi - \theta }^{\pi } \frac{{d^{2} \sin^{2} \varphi }}{{\sin^{2} \theta }}\left( {\mathop \int \limits_{{R\sin \left( {\pi - \theta } \right)}}^{R\sin \varphi } \frac{1}{{\left. {r^{2} } \right|_{y = h} }}dr} \right)d\varphi$$

for the water droplet with the apparent contact angle higher than 90°, and

5 $$t\frac{k\Delta T}{{\rho Ld}} = \mathop \int \limits_{\theta }^{0} \frac{{d^{2} \sin^{2} \varphi }}{{\sin^{2} \theta }}\left( {\mathop \int \limits_{R\sin \theta }^{R\sin \varphi } \frac{1}{{\left. {r^{2} } \right|_{y = h} }}dr} \right)d\varphi$$

for the water droplet with an apparent contact angle lower than 90°.

Where *d* is the diameter of the contact area between drop and substrate. The left-hand side of the equation is the non-dimensional freezing time in terms of the material properties of ice (*k*, $\rho ,L$) and measured freezing time *t*, and the right-hand side represents the term that can be solely determined by the final shape of the freezing drop and initial equilibrium contact angle.

**Figure 5:**
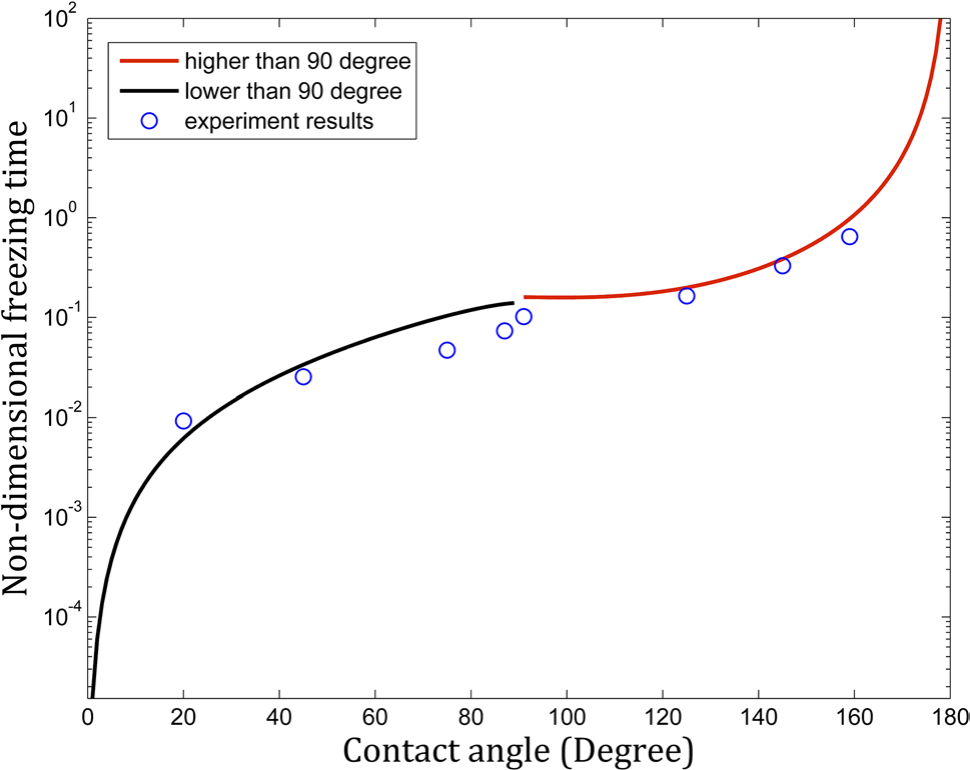
Theoretical calculation of the non-dimensional freezing time and the experiment results.

Figure 5 describes the theoretical calculation of the non-dimensional freezing time fitting against the experiment results and demonstrates the good agreement. The higher contact angle ensures a higher non-freezing time in a logarithm correlation. Samples in hydrophilic and hydrophobic ranges were both investigated and indicated the strong dependence on surface wettability. This can be explained qualitatively by the project contact area between the water droplet and the cold surface. The large contact area induces a significant high heat transfer rate and described quantitatively using our approach. Of course, the freezing time will be zero when liquid completely spreads out the surface and reaches infinity when the contact angle is 180 degrees (*d* = *0*). It should be noted here that we neglected convective effect inside the water droplet and the radiation between the water droplet and ambient air due to a short time experiment and main heat attributed to the conductive heat. Our calculation proposed the appropriate approach for calculating the freezing time of a sole water droplet on a sub-temperature substrate in a wide range of surface wettability and contributed to the designing of an icephobic surface.

In this conclusion, we proposed a facile method to prepare the superhydrophobic surface on Al plates for anti-icing purposes. The treated sample exhibited extremely high anti-icing performance in terms of adhesion strength and freezing time. Our results were compared with ongoing research works and demonstrated the relative agreement when high water contact angle and small work of adhesion ensured the low ice-surface adhesion. Furthermore, we presented a theoretical method to calculate the freezing time from the heat transfer approach. Experimental results were compared with theoretical prediction and described the good agreement, illustrating the correctness when considering the freezing time. This insight should lead to an understanding of icing phenomena and the design of icephobic surfaces.

## Methods

**Figure 6:**
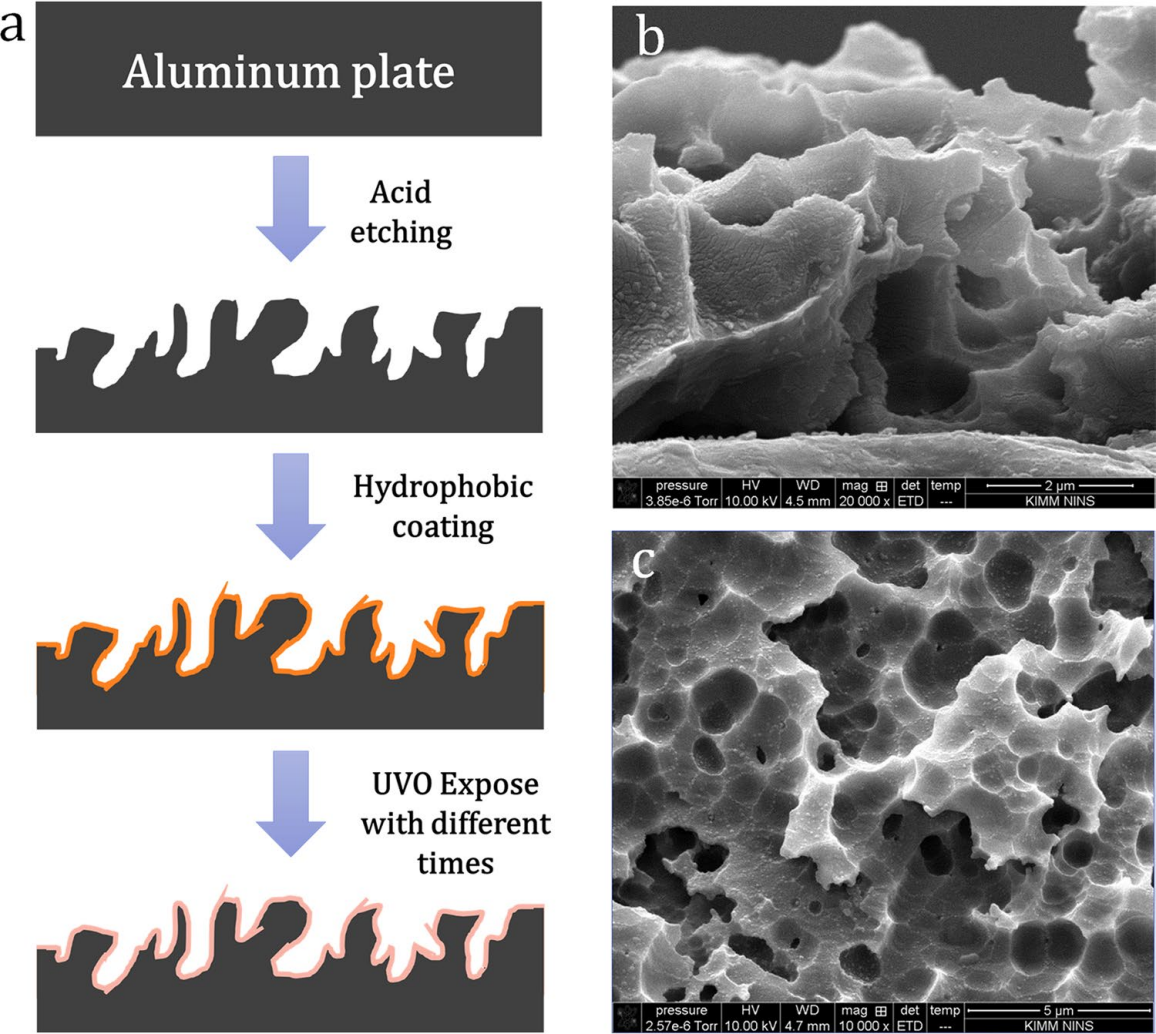
The fabrication process of examined samples (**a**) and SEM images of the surface after etching in cross-view (**b**) and top view (**c**). Reproduced with permission from Nguyen et al.^24^, © 2018 The Korean Society of Industrial and Engineering Chemistry.

The experiments were performed on Al plates produced in our previous work^24^, since they possess relatively high thermal conductivity and easy to make micro-nano hierarchical structures with tailored designs. Figure 6 describes the fabrication process and corresponded micro–nanostructure after the etching process. The hierarchical Al samples were achieved by a wet etching routine. After ultra-sonicated with Ethanol, Iso-Propanol (IPA) and Aceton in each 15 min, respectively, surfaces are dried with N_2_ flow and followed by dipping inside a solution contained HCl acid, IPA and DI water for 10 min at 200 °C. Etched samples were then immediately cleaned by Di-Water and drying by N_2_ flow. The hierarchical sample was then coated with FOTS (Fluoroctatrichlorosilane, Sigma-Aldrich Inc., Missouri, United States) via vapor phase coating for 1 h, followed by heating at 100 °C for another 1 h. After coating, the sample presented the perfect water repellent performance with an extremely high contact angle (159°) and a low sliding angle (below 2°). The other wettability was generated by Ultra Violet/Ozone (UVO) treatment with rational exposed time. The wettability of samples was determined using a contact angle measurement apparatus (Model DM-50, Kyowa Interface Science Co. Ltd., Saitama, Japan) with 5 μL deionized water droplets. The contact angle (CA) was averaged statistically with at least ten measurements in independent positions on each sample. The morphology details and structural information of all specimens are shown in Table 1.

**Table 1:** Structural information of examined samples.

No	Contact angle (°)	Work of adhesion (mN/m)	Adhesive strength (kPa)	Freezing time (s)	Note
#1	20	0.13966	1245	10	Superhydrophilic
#2	45	0.12291	1055	13	
#3	75	0.09663	958	15	
#4	90	0.072	580	25	
#5	120	0.0307	380	30	
#6	145	0.01302	230	43	
#7	159	0.00478	135	55	Superhydrophobic
#8	87	0.07577	1201	18	As-received Al

**Figure 7:**
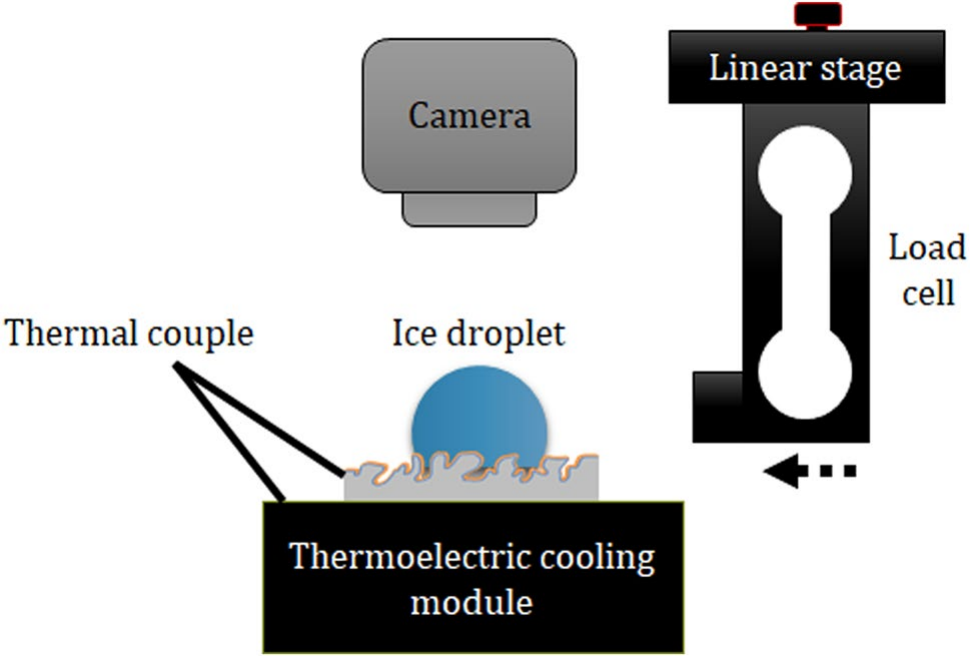
Experiment setup for measuring.

**Figure 8:**
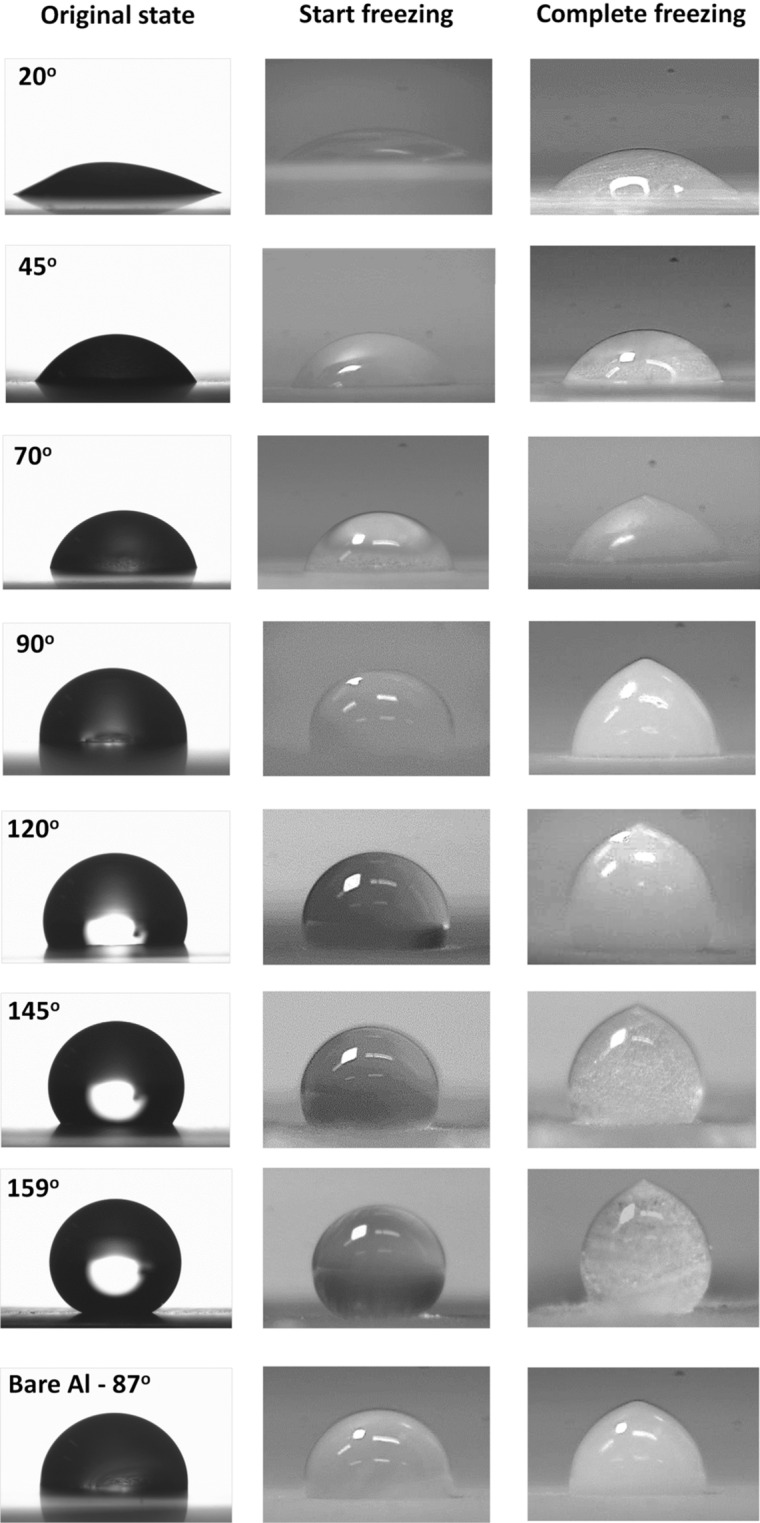
Icing formation on surfaces with different wettability observed by high-speed camera.

The ice-surface shear stress measurement was performed using a custom-built apparatus ^15,24^. Figure 7 describes the experimental setup model. Samples were carefully attached to a Peltier stage using the Al tape with high thermal conductivity. A 5uL deionized water drop was tenderly dropped onto the examined surface before started cooling. The cooling module temperature was maintained at − 10 °C to enable the freezing process. It should be noted here that the evaporation process was disregarded because of the short duration of the experiment (several minutes). A high-speed camera (Photron Ltd.) was used to observe the freezing process, the temperature evolution, and determined the freezing moment (Fig. 8). The freezing time is defined as the duration since phase transition occurs until it completes. After the freezing process ended i.e. water droplet completely solidified, the adhesion strength test took place using a force transducer load cell, which was connected to the movement apparatus and moving at a quite low speed (50 um/s) to horizontally push the ice droplet. The force exerted from collision slowly increased and displayed on computer software. The adhesive strength was defined as the maximum recorded value correspond to the moment ice bulk was detached from the surface.
