# Author Correction: Anti-icing performance on aluminum surfaces and proposed model for freezing time calculation

**DOI:** 10.1038/s41598-021-88230-7

**Published:** 2021-04-26

**Authors:** Van-Huy Nguyen, Ba Duc Nguyen, Hien Thu Pham, Su Shiung Lam, Dai-Viet N. Vo, Mohammadreza Shokouhimehr, Thi Hong Hanh Vu, Thanh-Binh Nguyen, Soo Young Kim, Quyet Van Le

**Affiliations:** 1 Department for Management of Science and Technology Development, Ton Duc Thang University, Ho Chi Minh City, Vietnam; 2 Faculty of Applied Sciences, Ton Duc Thang University, Ho Chi Minh City, Vietnam; 3 Basic Science Department, Tan Trao University, Tuyen Quang, Vietnam; 4 Surface Analysis Department, Samsung Display Vietnam, Bac Ninh, Vietnam; 5 Pyrolysis Technology Research Group, Higher Institution Centre of Excellence (HICoE), Institute of Tropical Aquaculture and Fisheries (AKUATROP), Universiti Malaysia Terengganu, 21030 Kuala Terengganu, Terengganu Malaysia; 6 Center of Excellence for Green Energy and Environmental Nanomaterials (CE@GrEEN), Nguyen Tat Thanh University, 300A Nguyen Tat Thanh, District 4, Ho Chi Minh City, 755414 Vietnam; 7 Department of Materials Science and Engineering, Research Institute of Advanced Materials, Seoul National University, Seoul, 08826 Republic of Korea; 8 Physics Faculty, Thai Nguyen University of Education, Thai Nguyen, Vietnam; 9 Department of Materials Science and Engineering, Institute of Green Manufacturing Technology, Korea University, 145 Anam-ro, Seongbuk-gu, Seoul, 02841 Republic of Korea; 10 Institute of Research and Development, Duy Tan University, Da Nang, 550000 Vietnam

Correction to: *Scientific Reports* 10.1038/s41598-020-80886-x, published online 11 February 2021

The original version of this Article contained errors.

The legend for Figure 6 did not acknowledge that the SEM images had been published previously. The text,

“The fabrication process of examined samples (**a**) and SEM images of the surface after etching in cross-view (**b**) and top view (**c**).”

Now reads,

“The fabrication process of examined samples (**a**) and SEM images of the surface after etching in cross-view (**b**) and top view (**c**). Reproduced with permission from Nguyen et al.^24^, © 2018 The Korean Society of Industrial and Engineering Chemistry.”

In addition, the text in the Methods,

“The experiments were performed on the Al plate since it possesses relatively high thermal conductivity and easy to make micro-nano hierarchical structures with tailored designs^24^.”

Now reads,

“The experiments were performed on Al plates produced in our previous work^24^, since they possess relatively high thermal conductivity and easy to make micro-nano hierarchical structures with tailored designs”.

In the original version of Figure 8, incorrect images were used for 20° Start freezing, 70° Start freezing, and 70° Complete freezing. The original version of Figure 8 appears below, as Figure 1.

**Figure 1:**
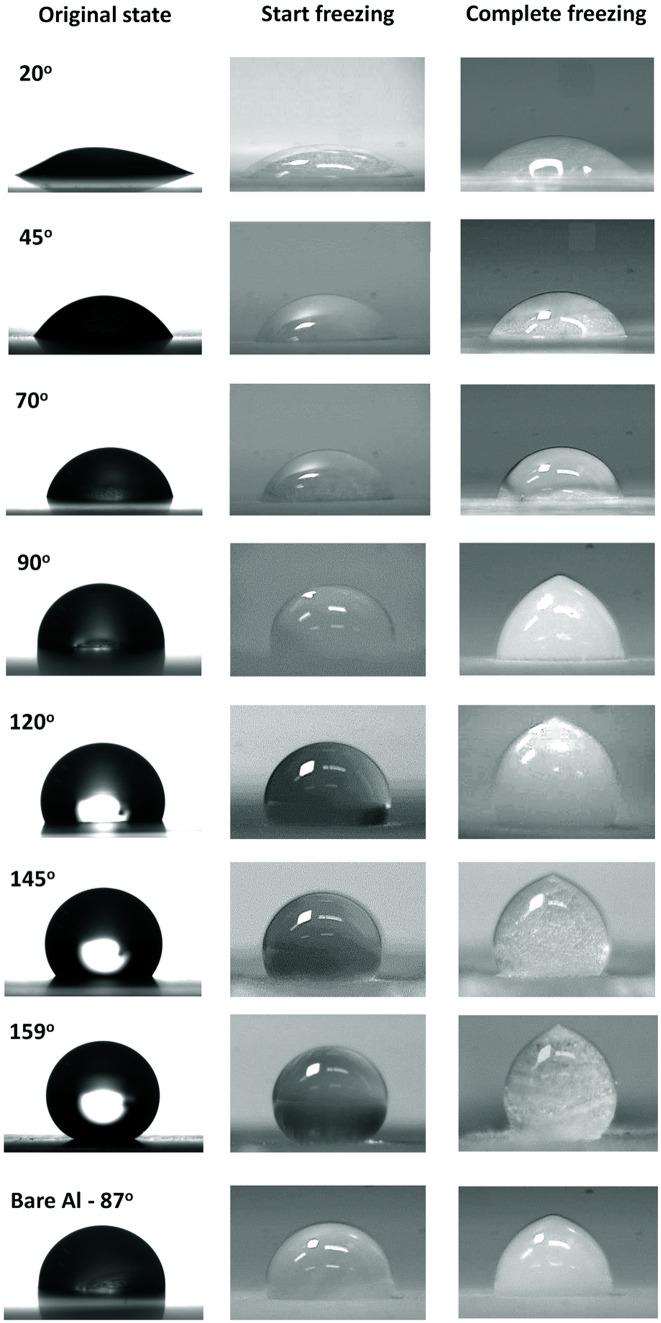
The original version of Figure 8.

These errors have now been corrected in the PDF and HTML versions of the Article.

